# Uptake and Transfer of a *Bt* Toxin by a Lepidoptera to Its Eggs and Effects on Its Offspring

**DOI:** 10.1371/journal.pone.0095422

**Published:** 2014-04-18

**Authors:** Débora Pires Paula, David A. Andow, Renata Velozo Timbó, Edison R. Sujii, Carmen S. S. Pires, Eliana M. G. Fontes

**Affiliations:** 1 Department of Biological Control, Embrapa Genetic Resources and Biotechnology, Brasília, DF, Brazil; 2 Department of Entomology, University of Minnesota, St. Paul, Minnesota, United States of America; 3 Department of Molecular Biology, University of Brasilia, Brasília, DF, Brazil; University of California, Berkeley, United States of America

## Abstract

Research on non-target effects of transgenic crop plants has focused primarily on bitrophic, tritrophic and indirect effects of entomotoxins from *Bacillus thuringiensis*, but little work has considered intergenerational transfer of Cry proteins. This work reports a lepidopteran (*Chlosyne lacinia*) taking up a *Bt* entomotoxin when exposed to sublethal or low concentrations, transferring the entomotoxin to eggs, and having adverse effects on the first filial generation (F1) offspring. Two bioassays were conducted using a sublethal concentration of toxin (100.0 ng/µl Cry1Ac) for adults and a concentration equal to the LC10 (2.0 ng/µl Cry1Ac) for larvae. Cry1Ac is the most common entomotoxin expressed in *Bt* cotton in Brazil. In the adult diet bioassay there was no adverse effect on the parental generation (P0) adults, but the F1 larvae had higher mortality and longer development time compared to F1 larvae of parents that did not ingest Cry1Ac. For the 3rd instar larvae, there was no measurable effect on the P0 larvae, pupae and adults, but the F1 larvae had higher mortality and longer development time. Using chemiluminescent Western Blot, Cry1Ac was detected in F_1_ eggs laid by P_0_ butterflies from both bioassays. Our study indicates that, at least for this species and these experimental conditions, a ∼65 kDa insecticidal protein can be taken up and transferred to descendants where it can increase mortality and development time.

## Introduction

Research on non-target effects of transgenic crop plants has focused primarily on understanding the environmental effects of entomotoxins from *Bacillus thuringiensis*, Cry proteins (crystalline proteins) and VIPs (vegetative insecticidal proteins), incorporated into *Bt* crops. Several major exposure-effects pathways have been extensively explored: direct or bitrophic exposure of the non-target species to the entomotoxin, either in its pure form in artificial diets or as it is expressed in plants, tritrophic exposure of the non-target species via consumption of a different species that fed directly on the pure entomotoxin or the *Bt* plant, and indirect, knock-on or cascading effects due to changes in the population density or behavior of another organism [1]–[4].

While it is widely agreed that Cry proteins can be transferred up the trophic chain [5], [6], there remains considerable uncertainty about how the transfer occurs. One possible way a prey can transfer Cry protein is to take up Cry protein in its body tissues and another non-mutually exclusive way is to fill its gut with food containing Cry protein [7], [8]. Only two studies have examined an internal tissue (hemolymph), and they did not detect Cry protein in it [9], [10].

It is also generally believed that the mode of action of Cry proteins is to bind to receptors in the peritrophic matrix of the midgut epithelial cells, forming pores and subsequently cell lysis [11], and facilitating the penetration of enteric bacteria into hemolymph, which induce septicemic death [12]. Most susceptible insects, however, show considerable variation in susceptibility to Cry proteins over various life stages. We investigated what might happen to these proteins when a susceptible lepidopteran species is exposed at sublethal or low concentrations during stages with low susceptibility. As defined by Desneux et al. [13], a sublethal dose is one that does not induce statistically significant mortality in the experimental population. We did not focus on potential chronic effects of these doses, as there is a wide literature on this topic [2], [4]. Instead, we focused on the possibility that these proteins could be taken up and transferred to other sites in the insect, such as eggs, where they could be detected and possibly have an effect on the first filial (F_1_) generation.

The vast majority of the compounds known to be taken up by larval and adult Lepidoptera have low molecular weight (<800 Da) [14]. These compounds are absorbed or endocytosed through the peritrophic matrix (PM) of the insect midgut, transported via the hemolymph and deposited in perivisceral fat body where they form multivesicular bodies inside the adipocyte and where they may be hydrolyzed [14]–[16]. Compounds that are secondary metabolites are known to be transported to the eggs in the “defense syndrome” of several Lepidoptera that protects eggs and neonates against natural enemies [17]–[21]. Although Cry proteins are much larger (120–130 kDa for a protoxin, and ∼65 kDa an activated form), the uptake and accumulation of a Cry protein (or at least the immunoreactive parts of Cry proteins) into the hemoceol has occurred for one species [22].

We used the nymphalid lepidopteran, *Chlosyne lacinia* (Geyer), in this study because it was the most abundant adult lepidopteran non-target flower visitor in cotton crops in the Brazilian Central West [23], and also is abundant in the agroecosystems of South and Central America [24], [25], feeding exclusively in the larval stage on Asteraceae leaves [26]. Consequently, we focused first on potential effects of nectar feeding on adult butterflies and their F_1_ offspring. The larvae and butterflies have vibrant conspicuous coloration [27], eggs are laid in clusters, and the larvae are gregarious up to the third instar; these are characteristics indicative of an aposematic species [28]. Many aposematic lepidopterans, particularly those with gregarious behavior in the immature stage [29], can sequester secondary metabolites of their host plants and transfer these metabolites to their eggs [15]. So, although exposure to larvae in cotton fields would be unlikely, we also investigated if in a low lethal concentrations of Cry protein (LC_10_) could be taken up by larvae and passed to their F_1_ offspring.

We fed Cry1Ac (activated form) protein to *C. lacinia* larvae and adults in independent experiments to investigate the uptake and transfer of Cry1Ac to eggs in this species. This entomotoxin is an appropriate model because it is the most commonly expressed Cry protein in Brazilian *Bt* cotton. The experiments aimed to address the following questions: (1) Can *C. lacinia* take up Cry1Ac protein into eggs when exposed to a sublethal or low concentration of toxin in the adult stage or in the larval stage? (2) If so, can the Cry1Ac protein in the eggs cause adverse effects to those F_1_ offspring?

## Materials and Methods

### *Chlosyne lacinia* colony rearing

The colony was continually resupplied with egg masses collected from leaves of the wild sunflower (*Tithonia diversifolia*, Asteraceae) in an experimental field at Embrapa Genetic Resources and Biotechnology, Brasilia - DF, Brazil. After hatching, the caterpillars were maintained at 25±2°C, 55±10% R.H. and 16 h photophase in 3.5 L plastic cages and fed daily with fresh leaves of hairy beggarticks (*Bidens pilosa*, Asteraceae) or sunflower (*Helianthus annuus*, Asteraceae). After adult emergence, the individuals were sexed and females were transferred to screened cages (1 m^3^) containing plants of dwarf-sunflower, and wild sunflower and hairy beggarticks. Egg masses were collected from the underside of the leaves and incubated in plastic cages in the same conditions that the larvae were reared.

### Verification of Cry1Ac receptors in the midgut

The midgut was dissected from 10 third instar larvae, 10 adult males and 10 adult females, and immersed in 900 µl of ice-cold SET buffer (250 mM sucrose, 17 mM Tris pH 7.5, 5 mM EGTA). Brush-border membrane vesicles (BBMV) were prepared by the differential magnesium precipitation method [30] as modified by Carroll and Ellar [31]. BBMV were centrifuged for 10 min at 13,500x*g* and resuspended in 100 µl binding buffer (25 mM Tris pH 7.5, 3 mM KCl, 135 mM NaCl) for binding assays. The Cry1Ac protein used in all experiments was produced by Dr. Pusztai-Carey (Department of Biochemistry, Case Western Reserve University, Cleveland, Ohio) and purchased purified and trypsinized to 64 kDa, like the active form expressed by Bt cotton. Cry1Ac (1 mg) was labeled with biotin using the protein biotinylation module (RPN 2202, GE Healthcare Life Science, USA) according to the manufacturer's protocol. Protein concentrations (BBMV and biotinylated Cry1Ac) were quantified by Bradford's method [32], using bovine serum albumin (BSA) as the standard. The binding assays were conducted by incubating 10 nM biotinylated Cry1Ac with 10 µg BBMV in 100 µl of binding buffer for 1 h at room temperature. After incubation, samples were centrifuged at 16,000x*g* for 10 min. The pellets were suspended in 10 µl of electrophoresis sample buffer and samples were fractionated using 10% sodium dodecyl sulfate-polyacrylamide gel electrophoresis (SDS-PAGE). Fractionated proteins were electrotransferred to a 0.45 µm polyvinylidene fluoride (PVDF) membrane (Hoefer, USA). Receptors bound to biotinylated Cry1Ac were detected by Enhanced Chemiluminescence (ECL) Western Blotting according to manufacturer's instructions (RPN 2202, GE Healthcare Life Science, USA) using streptavidin conjugated to peroxidase (dilution 1∶1,500), adding luminol (SuperSignal West Pico Chemiluminescent Substrate, Pierce Thermo Scientific, USA) as recommended by the manufacturer, and exposing X-ray film (Amersham Hyperfilm ECL, GE Healthcare Life Science, USA) for 10 min.

### Cry1Ac concentration in *Bt* cotton nectar

Since the release of *Bt* Bollgard cotton for commercial planting in 2005 in Brazil [33], the area of *Bt* cotton grew rapidly, mainly in the Central West Region of Brazil [34]. Based on this information, *Bt* cotton (variety DeltaPine Bollgard NuOPAL) was chosen as a reference for determining the toxin dosage that an adult butterfly could be exposed to in the field through nectar feeding. Quantification of Cry1Ac expression in nectar was made by ELISA. To obtain cotton nectar, 70 plants each of the Bt cotton variety (DeltaPine Bollgard DeltaOPAL) and a near isogenic non-Bt variety were grown in the greenhouse in 350 ml pots. Nectar from the near isogenic non-Bt cotton plants was used as the blank in the quantification of Cry1Ac. Plants were watered daily in the morning and fertilization was applied biweekly (10∶10∶10 NPK and micronutrients) at recommended rates. After four months, the cotton plants were transplanted into larger pots (4 l) to maximize flower production for nectar collection.

For several days, nectar was collected from 600 flowers with a sterile micropipette and stored in microtubes of 1.5 ml and frozen at −80°C until molecular analysis. The total nectar (around 500 µl for all flowers) was centrifuged for 20 min at 10,000xg at 4°C and the supernatant collected for ELISA and for butterfly bioassay. Cry1Ac quantification was performed with a *Bt*1Ac SDI kit (Gehaka), according to the manufacturer's manual, using the Cry1Ac standards of 0, 5.0, 10.0, 15.0, 20.0, 25.0, 30.0, 35.0, 40.0, 45.0 and 50.0 ng/well, replicated three times for the calibration curve, and the *Bt* and non-*Bt* nectar samples were also replicated three times. The absorbance was read at 450 nm. The average quantity of Cry1Ac in the *Bt* cotton nectar per 5 µl nectar was estimated with the linear regression equation of the Cry1Ac standards (ng) on their average absorbance, and the average absorbance of the Bt nectar sample minus the absorbance of the non-Bt nectar blank.

### Lifetime nectar consumption by adults

**Figure 1:**
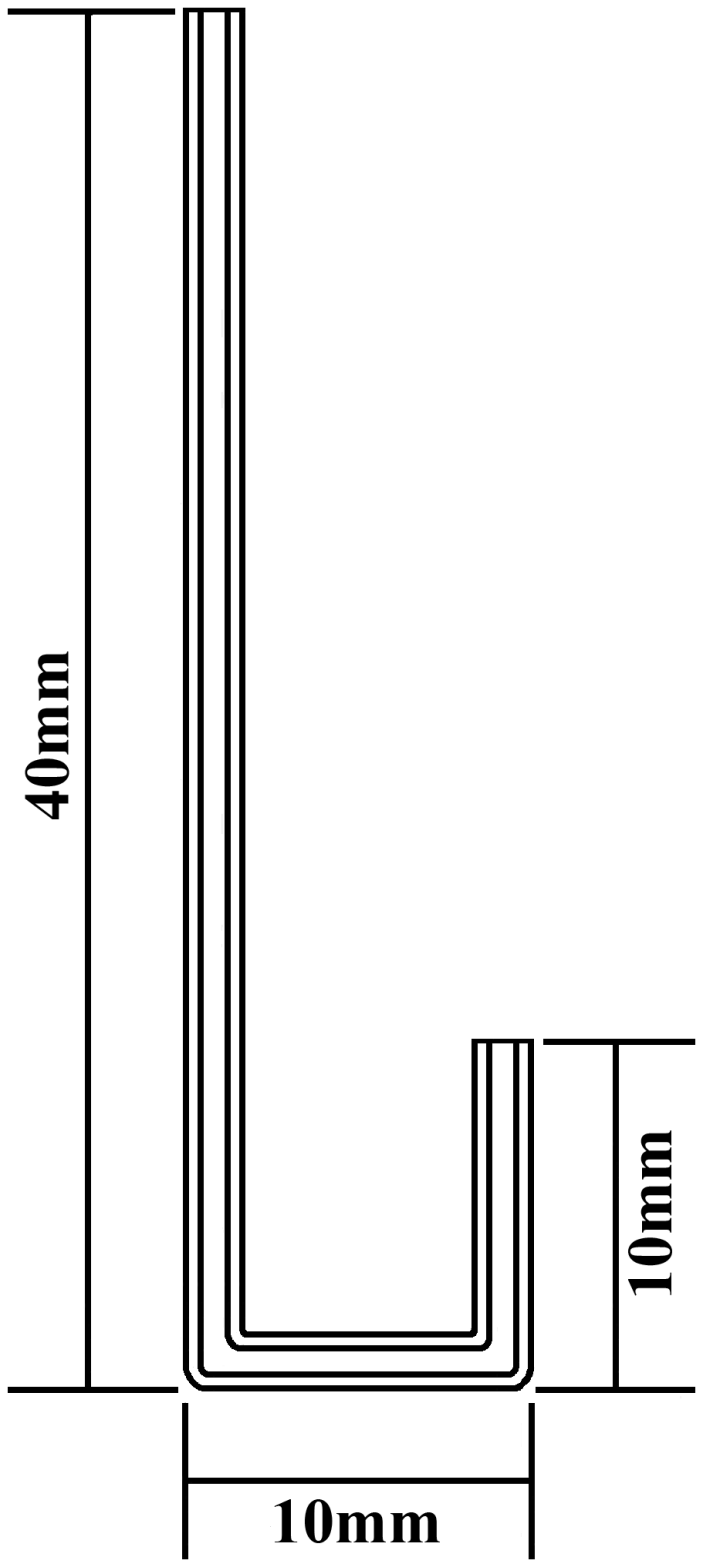
Illustration of microcapillary tube used to estimate lifetime adult feeding.

The average lifetime diet volume ingested per butterfly was estimated for each sex by offering artificial nectar, 30% (v/v) honey, in a 100 µl microcapillary tube. The microcapillary tube was double folded on a Bunsen burner (Figure 1) and placed in an artificial flower that mimicked a nectar host flower (hairy beggarticks). The volume of diet ingestion was estimated by multiplying the decreased height (mm) in the microcapillary tube by cross-sectional area (mm^2^) (calculated by the microcapillary tube diameter) minus the evaporation measured in this feeding system. Thirty freshly emerged unmated adults of each sex were fed diet individually in 30×30×50 cm cages. Microcapillary tubes were replaced every day until death. Diet consumption by male and female butterflies was compared with a Student's *t*-test.

### Adult diet bioassay for toxicity of Cry1Ac

The adult diet bioassay consisted of three treatments applied to parental generation (P_0_) adults: T0: Negative control (30% v/v honey); T1: Positive control (100.0 ng/µl casein added to 30% v/v honey); and T2: *Bt*-treatment (100.0 ng/µl Cry1Ac added to 30% v/v honey). The *Bt* treatment applied Cry1Ac at ∼140 times the concentration found in NuOPAL cotton nectar, as it is common in ecotoxicology studies to expose the target species to several hundred times the expected field concentration [35]. Experiments were conducted with 100 recently emerged P_0_ butterflies in each treatment (sex ratio 1∶1), and each experiment was replicated three times. The purpose of the casein treatment (T1) was to test the influence of a protein in the butterfly liquid diet [36]. Adult butterflies do not have proteinases, and dietary protein can reduce their survival. Every day 10 µl of the diet was applied on natural flowers of hairy beggartick inside the cages containing butterflies. Ten P_0_ butterflies (from each sex) and 10 F_1_ egg masses from each treatment were collected on the fourth day and stored at −20°C for later molecular analysis. The following responses were measured on the P_0_ adults: adult longevity (days), number of egg masses laid, egg mass size. Longevity was measured for each adult and averaged within each cage for statistical analysis. In addition, from each replicate cage, F_1_ eggs were collected so that 100 F_1_ neonate larvae (N = 300 total for each treatment) were used to measure development time (days) and survivorship (%) to pupation in the F_1_ generation. Development time was measured on each of the 100 larvae and averaged within replicate for subsequent statistical analysis. Finally, 10 first instar F_1_ larvae were taken from each treatment and each replicate of the experiment and stored at −20°C for later molecular analysis.

### LC_10_ determination for leaf dip bioassay for larvae

We estimated the LC_10_ of the Cry1Ac toxin to provide a toxin exposure that would allow larval development and subsequent reproduction. The LC_10_ was estimated through probit analysis after Abbott's correction [37] using 10 larvae of each instar per toxin concentration replicated three times, exposed to 0, 1.0, 2.0, 5.0, 10.0 and 20.0 ng/µl of Cry1Ac in Tween 0.02% solution. The larvae had molted within the past 24 h, when they were transferred into plastic pots (350 ml) containing wild sunflower leaves freshly immersed in the experimental solutions for 30 s. Excess solution on the leaves was drained by putting the leaves on paper towels for 5 min. The area of wild sunflower leaf provided was constant within each instar, but more leaf tissue was provided to older instars. The treated wild sunflower leaves were renewed every day. The mortality rate was recorded on the fifth day, by which time surviving larvae had molted to the next instar.

### Leaf dip bioassay for toxicity of Cry1Ac to larvae

The leaf dip toxicity bioassay consisted of two treatments applied to P_0_ 3^rd^ instars: T0 - Control group (Tween 0.02% solution) and T1 - Cry1Ac at 2.0 ng/µl in Tween 0.02% solution, with 100 P_0_ 3^rd^ instars in each treatment. Experiments were replicated three times. Within each experiment, groups of 10 P_0_ larvae were transferred onto plastic pots (500 ml) containing wild sunflower leaves freshly immersed in the experimental or control solution for 30 s and drained of excess solution on paper towels for 5 min. The wild sunflower leaf dishes were renewed every day. Faeces were collected from P_0_ larvae every day (average of 10 mg) and immediately stored at −20°C for later molecular analysis. The mortality rate was recorded on the fourth day, by which time surviving P_0_ larvae had molted to the 4^th^ instar. On the fifth day, sunflower leaves without any treatment were provided to the surviving P_0_ larvae, and after 24 h, one P_0_ larva from each group in a replicate per treatment (*N* = 30 total for each treatment) was collected and stored at −20°C for later molecular analysis. The others were reared to the adult stage, mated and let to lay eggs. One P_0_ pupae, one P_0_ butterfly (of each sex), one F_1_ egg mass, and one F_1_ 1^st^ instar from each group in a replicate per treatment (*N* = 30 total for each treatment) were also collected and stored at −20°C for later molecular analysis. The following responses were measured on each group of 10 larvae in the P_0_ generation: larval and pupal development time (days) and survivorship (%), adult longevity (days), and reproductive performance. Development time and longevity were measured on each individual and averaged within groups. F_1_ eggs were collected from the P_0_ adults so that for each treatment and each experimental replicate 100 F_1_ neonate larvae were used to measure development time (days) and survivorship (%) to pupation in the F_1_ generation (N = 300 larvae total for each treatment). Development time was measured on each of the 100 larvae and averaged within replicates for subsequent statistical analysis.

### Cry1Ac detection in *C. lacinia*

All the samples collected in both bioassays were individually weighed and homogenized in 1.5 ml microtubes with 50 mM TrisHCl buffer pH 7.0 with 0.05% Triton X-100, 0.2 M EDTA, and 1 mM PMSF in the proportion of 200 µl buffer per 10 mg of sample. The suspension was homogenized for 30 s on a vortex mixer and centrifuged at 5000x*g* for 15 min to remove particulate matter. The supernatants (10 µl) were used for chemiluminescent (ECL) Western Blotting and ELISA toxin detection. In the first methodology, the supernatants were fractionated in gradient SDS-PAGE from 5 to 20%, and the ECL was conducted by the same methodology described earlier, except that the anti-Cry1Ac antibody was biotin-labelled and diluted 1∶500. The limit of detection (LOD) of the ECL Western Blotting is reported by the manufacturer to be at least as low as 10 pg. The ELISA was performed using the Bt1Ac SDI kit (Gehaka), according to manufacturer manual, using the Cry1Ac standards for the calibration curve of 0, 1.0, 2.5, 5.0, 10.0, 25.0, and 50.0 ng/well replicated three times and the samples were also replicated three times. The absorbance reading was at 450 nm. The average level of toxin per mg of the corresponding insect samples was estimated from the linear regression equation of the Cry1Ac standards (ng) on their average absorbance, subtracting the absorbance of the blank. The LOD of the ELISA is reported to be at least as low as 1 ng, about 100x less sensitive than the ECL Western Blot.

### Statistical analyses of bioassay results

Either ANOVA (adult diet bioassay) or Student's *t*-test (leaf-dip bioassay) were used to analyze adult longevity (days), number of egg masses laid, number of eggs per egg mass, and immature development time (days). Treatment means were separated by Tukey's HSD for significant ANOVA results. Percent survival and number of eggs hatched were analyzed by GLM with binomial error. The adult diet bioassay results were analyzed as a single factor ANOVA with three replicates. The LC_10_ leaf-dip bioassay was analyzed as a two-factor, complete factorial ANOVA with three replicates, The P_0_ results from the leaf-dip bioassay were analyzed using the 30 larval groups as replicates. The F_1_ results from the leaf-dip bioassay were analyzed with the three experiments as replicates.

## Results

### Verification of Cry1Ac receptors in the midgut

**Figure 2:**
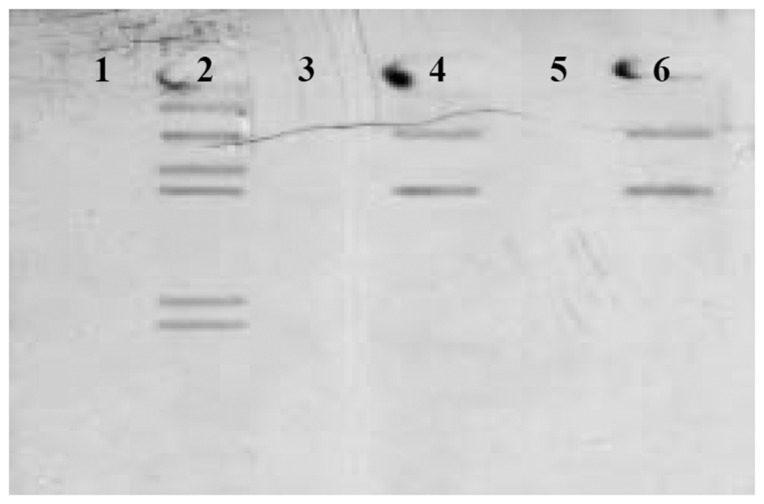
Cry1Ac ligand blot receptors in the lepidopteran *Chlosyne lacinia* isolated from midgut BBMV. For each lane 10 µg of BBMV were used (*N* = 10 individuals). Lanes: 1. Larval BBMV without binding assay with biotinylated-Cry1Ac (control-group); 2. Larval BBMV binding assay with biotinylated-Cry1Ac; 3. Male butterfly BBMV without binding assay with biotinylated-Cry1Ac (control-group); 4. Male butterfly BBMV with binding assay with biotinylated-Cry1Ac; 5. Female butterfly BBMV without binding assay with biotinylated-Cry1Ac (control-group); and 6. Female butterfly BBMV with binding assay with biotinylated-Cry1Ac.

Third instar larvae and male and female *C. lacinia* butterflies have Cry1Ac receptors in the midgut (Fig. 2). There is no dimorphism for Cry1Ac receptors between male and female adults, however they have a reduced expression of Cry1Ac receptors compared to third instar larvae (Fig. 2).

### Cry1Ac in cotton nectar, nectar consumption, and adult bioassay

The concentration of Cry1Ac in *Bt* cotton nectar (Bollgard DeltaPine NuOPAL) was estimated by ELISA to be 0.72±0.10 ng Cry1Ac/µl of nectar. A butterfly imbibed 12±3 µl nectar in its lifetime, so an adult in the bioassay consumed 1200.0±300.0 ng Cry1Ac in their lifetime. No significant difference in diet consumption between males and females was observed (*t*_58_ = 1.89, *P* = 0.069). There were no detrimental effects of the casein protein on any of the P_0_ adult parameters measured (Table 1). In addition, despite the high concentration, there were no detrimental effects of Cry1Ac on either P_0_ male or female adult C. *lacinia*.

**Table 1:** Adult diet bioassay with Cry1Ac on adult *Chlosyne lacinia* and their offspring.

	Diet treatments			
Biological parameters studied	Honey 30%	Honey 30% + casein	Honey 30% + Cry1Ac	*P-value*
	(Control)	(100 ng/µl)	(100 ng/µl)	
*1. Cry1Ac adult bioassay*				
1.1 Sample size (N)	288	291	293	-
1.2 Longevity (days)	3.7±1.7 a^*^	3.8±1.9 a	3.8±1.3 a	ns;^†^ ANOVA
1.3 Reproductive performance				
1.3.1 Egg masses/female	2.8±1.1	2.9±1.0	3.1±1.2	ns; ANOVA
1.3.2 Eggs/egg mass	147.0±16.7 a	153.4±10.8 a	150.3±11.3 a	ns; ANOVA
1.3.3 Eggs hatched (%)	78.8±5.1 a	69.9±7.5 a	81.8±5.3 a	ns, GLM
*2. F_1_ Offspring larvae*				
2.1 Sample size (N)	300	300	300	-
2.2 Development time (days)	18.9±3.2 a	17.9±3.3 a	30.1±6.3 b	0.008; ANOVA, Tukey
2.3 Survivorship (%)	88.7 a	87.0 a	12.7 b	0.009; GLM

*The means are followed by standard deviations. Values followed by the same letter are not significantly different.

^†^ns: not significantly different.

Detrimental effects of Cry1Ac were observed in the F_1_ offspring of the Cry1Ac-treated P_0_ butterflies. F_1_ larvae whose parents ingested Cry1Ac showed a significantly retarded larval development time (from 18.9 to 30.1 days, increase of 1.6 times) and lower survivorship (from 88.8 to 12.8%, almost seven times less).

### Cry1Ac leaf dip bioassay with third instars

The Cry1Ac LC_10_ for the leaf dip bioassay was determined to be 2.0 ng/µl for the third instar (Table 2). The first and second instars were more sensitive to the toxin at all concentrations tested. Third, fourth and fifth instar caterpillars were similarly affected by Cry1Ac, however as the gregarious feeding behavior of the larvae occurs in the first two instars, the third instar was used in these experiments.

**Table 2:** Mortality (%) of *Chlosyne lacinia* (Lepidoptera: Nymphalidae) larvae exposed to Cry1Ac on wild sunflower leaves after immersion for 30 s in a solution containing the indicated concentration of Cry1Ac and 0.02% Tween (*N* = 10 larvae per instar for each toxin concentration, and were replicated three times).

Cry1Ac (ng/µl)	Instar				
	First	Second	Third	Fourth	Fifth
0.0	3.9 a,a^*^	2.8 a,b	2.0 a,c	5.0 a,d	11.0 a,e
1.0	26.2 b,a	17.7 b,b	8.0 b,c	9.6 b,c	12.4 a,c
2.0	35.0 c,a	22.5 b,b	10.1 b,c	12.0 b,c	14.0 a,c
5.0	65.4 d,a	52.0 c,b	24.3 c,c	24.1 c,c	23.9 b,c
10.0	82.0 e,a	71.6 d,b	49.9 d,c	37.7 d,d	28.1 b,e
20.0	84.9 e,a	74.0 d,b	60.8 e,c	51.2 e,d	45.0 c,d

*Values followed by the same letter are not significantly different. The first letter indicates the difference in the same column (related to the mortality in each Cry1Ac concentration in one instar) and the second letter indicates the difference in the same line (related to the mortality in one concentration of Cry1Ac among the instars). The comparison was made by GLM (P<0.01).

Exposure to 2.0 ng/µl Cry1Ac during the third instar did not affect any of the response variables of the P_0_ caterpillars (Table 3). All larval, pupal, and adult P_0_ parameters measured were not significantly affected by Cry1Ac at this low exposure, so this concentration had no detectable toxicological effect. However, F_1_ larval offspring of exposed P_0_ larvae had a longer development time (around 1.7 times longer) and lower survivorship (around 4 times less) than F_1_ offspring from control P_0_ larvae (Table 3).

**Table 3:** Leaf dip bioassay with Cry1Ac on 3^rd^ instar larvae of *Chlosyne lacinia*, and surviving pupae and adults and their offspring.

Biological parameters studied	Leaf treatments		*P-value*
	− Cry1Ac	+ Cry1Ac	
	(Control)	(2.0 ng/µl)	
*1. Larvae*			
1.1 Sample size (*N*)	300	300	-
1.2 Development time (days)	4.0±1.0^*^	3.7±1.2	ns^†^; Student's *t-*test
1.3 Survivorship (%)	89.0	81.0	ns; GLM
*2. Pupae*			
2.1 Sample size (*N*)	267	241	-
2.2 Development time (days)	5.4±1.3	5.2±1.7	ns; Student's *t-*test
2.3 Survivorship (%)	75.3	71.3	ns; GLM
*3. Adults*			
3.1 Sample size (*N*)	200	193	-
3.2 Longevity (days)	4.1±1.3	4.0±1.3	ns; Student's *t-*test
3.3 Reproductive performance			
3.3.1 Egg masses/female	3.0±1.3	2.9±0.9	ns; Student's *t-*test
3.3.2 Eggs/egg mass	171.9±15.8	163.8±12.6	ns; Student's *t-*test
3.3.3 Egg hatched (%)	81.1±5.3	78.4±6.9	ns; GLM
*4. F_1_ Offspring larvae*			
4.1 Sample size (*N*)	300	300	-
4.2 Development time (days)	17.7±3.0	30.7±7.7	0.006; Student's *t-*test
4.3 Survivorship (%)	85.0	11.7	0.005; GLM

*The means are followed by standard deviations.

^†^ns: not significantly different.

### Cry1Ac detection in *C. Lacinia*

**Figure 3:**
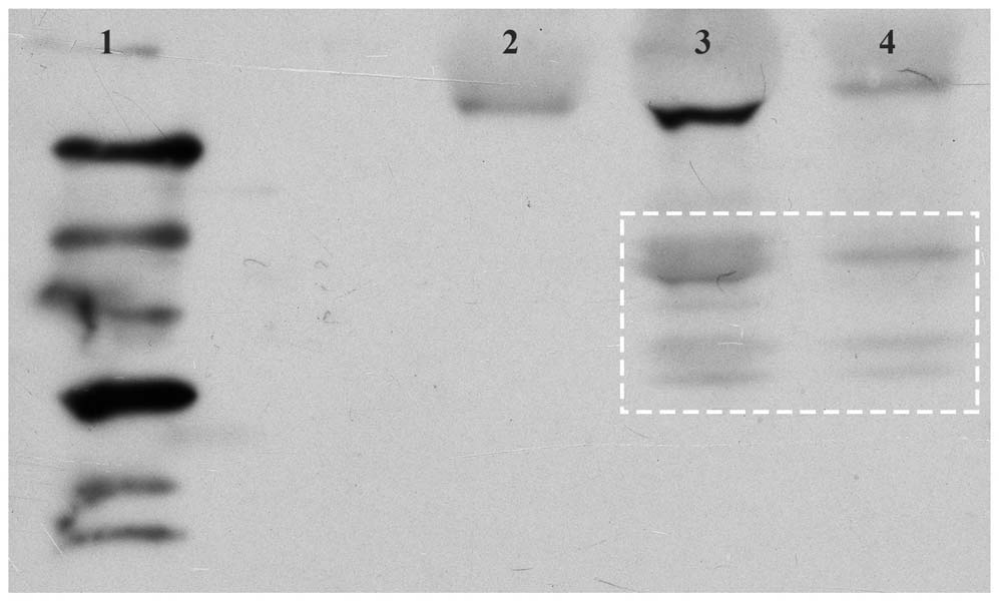
Cry1Ac detection (dashed square) in the egg masses of the lepidopteran *Chlosyne lacinia* by ECL Western Blot. Lanes: 1. Biotin Amersham ECL Ladder (GE Healthcare Life Science, USA); 2. One F_1_ egg mass corresponding to around 100 eggs from the control group (parents without exposure to Cry1Ac); 3. One F_1_ egg mass from P_0_ adults exposed to Cry1Ac protein in the adult diet bioassay; 4. One F_1_ egg mass from P_0_ parents exposed to Cry1Ac protein in the third instar in the leaf dip bioassay.

Cry1Ac was detected by ELISA in larval faeces 24 h (0.05 ng Cry1Ac/mg faeces) and 72 h (9.43 ng Cry1Ac/mg faeces) from the start of the larval exposure experiment. It was also detected by ECL Western Blot in the egg masses obtained in both the adult and larval exposure experiments (Fig. 3), demonstrating that the Cry1Ac ingested in the adult phase (shortly after emergence) or in the immature phase (third instar) was taken up and transferred to eggs. As evidenced by the bands smaller than 65 kDa, some proteolysis or degradation of the Cry1Ac protein had occurred. The band larger than 97 kDa that occurred in all treatments was an indication of endogenous biotinylated protein(s). The toxin was detected as traces by ELISA in female butterflies and in the offspring of both adults and larvae subjected to Cry1Ac treatments.

## Discussion

Adult and larval *C. lacinia* consuming sublethal and LC_10_, respectively, quantities of Cry1Ac took up and transferred activated Cry1Ac to their eggs, and this caused lower survivorship and longer development time in the F_1_ larvae. Our results showed that Cry1Ac can be intra-specifically (transovarially) transferred, and corroborate the Zhang et al. [22] study, which showed that Cry1Ac was taken up from aphids feeding on Bt cotton and transferred by the coccinellid predator *Propylea japonica* to its eggs. Our results extend their findings to include effects on the F_1_ larval offspring.

Cry1Ac receptors are commonly found in the midgut of lepidopteran larvae [38], but few studies have looked for receptors in adult Lepidoptera, and our finding of midgut receptors in adults is a rare report. Adults appear to express a subset of the receptors expressed by the larvae. Despite the expression of receptors, adults are not susceptible to Cry1Ac, possibly because not all binding results in pore-formation [39]. In any event, the differential expression of receptors between the larval and adult stages might contribute to the loss of the susceptibility by adults even at high Cry1Ac protein concentrations.

The physiological mechanism, by which uptake occurs, is not yet known and its elucidation is beyond the scope of this work. However once it crossed the peritrophic matrix, the Cry protein might be stored in the perivisceral fat body [40], [41] like other proteins, and from there it might be transported to oocytes through a nonselective process or in association with the vitellogenin transport process, as already reported for other unusual proteins [42]–[44].

Our results indicate that a large protein can be taken up and transferred inter-generationally, and may suggest the possibility of greater interaction between environmentally available proteins and insect autecology. Despite the hundreds of studies on the effects of Cry proteins on non-target arthropods, none have looked for effects on the F_1_ generation. Only one study [22] tried to detect Cry protein in the F_1_ generation and they detected a Cry protein in the eggs of Propylea japonica (Coleoptera: Coccinellidae), so it is possible that such inter-generational transfer may be underappreciated.

We consider C. *lacinia* to be a model system for understanding effects of high concentrations of Cry proteins on non-susceptible adults and their F_1_ offspring, and LC_10_ on more tolerant larval stages and their F_1_ offspring. In the field, adult C. lacinia are exposed to Cry protein because they are the most common lepidopteran visitor to Bt cotton in Brazil, and larvae of several species of non-target Lepidoptera may be exposed to a lethal or sublethal concentration of Cry protein, because Cry protein concentrations in commercial Bt cotton vary considerably both spatially and temporally with a marked decline during flowering and boll maturation [45]. However it must be emphasized that risk assessment studies should integrate these laboratory results within an ecological context before speculating on the ecological risk of intergenerational transfer of Cry1Ac protein on species diversity and ecological services provided by non-target organisms in agroecosystems.
